# Predictive factors of romiplostim response in patients with refractory aplastic anemia: data from two clinical trials

**DOI:** 10.1007/s00277-025-06337-7

**Published:** 2025-07-01

**Authors:** Jun Ho Jang, Kinuko Mitani, Yoshiaki Tomiyama, Koji Miyazaki, Koji Nagafuji, Kensuke Usuki, Nobuhiko Uoshima, Tomoaki Fujisaki, Hiroshi Kosugi, Itaru Matsumura, Ko Sasaki, Masahiro Kizaki, Masashi Sawa, Michihiro Hidaka, Naoki Kobayashi, Satoshi Ichikawa, Yuji Yonemura, Kenta Murotani, Mami Shimizu, Akira Matsuda, Keiya Ozawa, Shinji Nakao, Jong Wook Lee

**Affiliations:** 1 Department of Hematology, Sungkyunkwan University Samsung Medical Center, Seoul, Republic of Korea; 2 Department of Hematology and Oncology, Dokkyo Medical University, Tochigi, Japan; 3 Department of Blood Transfusion, Osaka University Hospital, Osaka, Japan; 4 Department of Transfusion and Cell Transplantation, Kitasato University School of Medicine, Sagamihara, Japan; 5 Department of Hematology, Kurume University Hospital, Kurume, Japan; 6 Department of Hematology, NTT Medical Center Tokyo, Tokyo, Japan; 7 Department of Hematology, Japanese Red Cross Kyoto Daini Hospital, Kyoto, Japan; 8 Department of Internal Medicine, Matsuyama Red Cross Hospital, Matsuyama, Japan; 9 Department of Hematology, Ogaki Municipal Hospital, Ogaki, Japan; 10 Department of Hematology and Rheumatology, Faculty of Medicine, Kindai University, Osaka, Japan; 11 Department of Hematology, Saitama Medical Center, Saitama Medical University, Saitama, Japan; 12 Department of Hematology and Oncology, Anjo Kosei Hospital, Anjo, Japan; 13 Department of Hematology, National Hospital Organization Kumamoto Medical Center, Kumamoto, Japan; 14 Department of Hematology, Sapporo Hokuyu Hospital, Sapporo, Japan; 15 Department of Hematology, Tohoku University Hospital, Sendai, Japan; 16 Department of Transfusion Medicine and Cell Therapy, Kumamoto University Hospital, Kumamoto, Japan; 17 Biostatistics Center, Kurume University, Kurume, Japan; 18 Kyowa Kirin Co., Ltd, Tokyo, Japan; 19 Department of Hemato-Oncology, Saitama Medical University International Medical Center, Saitama Medical University, Saitama, Japan; 20 Division of Hematology, Jichi Medical University, Tochigi, Japan; 21 Division of Hematology/Respiratory Medicine, Faculty of Medicine, Institute of Medical Pharmaceutical and Health Sciences, Kanazawa University, Kanazawa, Japan; 22 Division of Hematology-Oncology, Hanyang University Seoul Hospital, 222-1 Wangsimni-Ro, Seongdong-Gu, 04763 Seoul, Republic of Korea

**Keywords:** Aplastic anemia, Refractory, Romiplostim, Hematological response, Predictor, Reticulocyte

## Abstract

The thrombopoietin receptor agonist (TPO-RA) romiplostim has been shown to be efficacious in the treatment of aplastic anemia (AA) refractory to immunosuppressive therapy. However, there are few reports on predictors of TPO-RA efficacy. We analyzed patient clinical and baseline laboratory data from two clinical trials that enrolled patients with refractory AA, a phase 2 dose-finding study (NCT02094417) and a phase 2/3 study (NCT02773290), to determine predictors of romiplostim efficacy. Data from 66 patients (35 from the phase 2 dose-finding study and 31 from the phase 2/3 study), median age 47.0 (range 20–78) years, were analyzed. Of these, 51.5% (34/66) had non-severe AA, 86.4% (57/66) had received prior therapy with anti-thymocyte globulin plus cyclosporine, and 80.3% (53/66) were transfusion-dependent. At 27 weeks, no patients achieved a complete response, 57.4% (35/61) achieved a partial response, 49.0% (24/49) became red blood cell transfusion-independent, and 56.8% (21/37) became platelet transfusion-independent. Univariate logistic regression analysis identified duration from diagnosis (*P* = 0.040), baseline reticulocyte count (*P* < 0.001), and platelet count (*P* < 0.001) as predictors of response to romiplostim at 27 weeks. Receiver operating characteristic curve analysis identified a cutoff value of 30.77 × 10^9^/L at baseline as the threshold reticulocyte count to predict response to romiplostim in patients with refractory AA (sensitivity: 82.9%, specificity: 73.1%). Of note, the initial dose included in this analysis was lower than that used in real-world clinical practice, as data from the phase 2 dose-finding study were used. This study identified factors predicting response to romiplostim at 27 weeks.

Trial registration: ClinicalTrials.gov NCT02094417 and NCT02773290.

**Supplementary Information:**

The online version contains supplementary material available at 10.1007/s00277-025-06337-7.

##  Introduction

Aplastic anemia (AA) is a rare, life-threatening disease characterized by progressive pancytopenia, primarily due to bone marrow failure [1, 2]. AA is caused by an autoimmune mechanism in which bone marrow function is inhibited by an abnormal T-cell response triggered by a variety of factors. The cause of the disease is mostly acquired and idiopathic, with only a small percentage of cases having a congenital genetic predisposition [3]. Once patients are suspected of having AA, rapid and accurate diagnosis and concomitant supportive care are important. Current treatment guidelines recommend the use of thrombopoietin (TPO) receptor agonists (TPO-RAs), immunosuppressive therapy (IST), and hematopoietic stem cell transplantation [4, 5].

Romiplostim is a TPO-RA recently approved for the treatment of AA in 2019 in Japan based on the results of a phase 2 dose-finding study [6] and a phase 2/3 study on its efficacy and safety [7]. Recent approvals for this indication have also been granted in other Asian countries including South Korea, Taiwan, Singapore, Malaysia, and Thailand. In the phase 2 dose-finding study, 10 (30%) patients achieved a platelet response at week 9, with seven of these patients (70%) being from the 10 μg/kg dose group. In the extension part of the phase 2 dose-finding study, 18 (55%) patients had a response, and 10 (30%) maintained the response for 2–3 years. Erythroid and neutrophil responses were also observed [6]. Based on the results of the phase 2 dose-finding study, the phase 2/3 study was conducted with a starting dose of 10 µg/kg and a maximum dose of 20 µg/kg of romiplostim. In the phase 2/3 study, 84% of patients experienced any hematological response (improvement in one of the three lines) after 27 weeks of treatment, and 39% of patients achieved a trilineage response after 53 weeks of treatment. The treatment was safe and well tolerated [7].

Predictors of the efficacy of the oral TPO-RA eltrombopag in AA refractory to IST have been reported to be higher absolute reticulocyte counts and shorter duration from the first IST [8–10]. A higher baseline reticulocyte count can significantly enhance the efficacy of eltrombopag [8, 9]. However, the predictors of the efficacy of romiplostim in refractory AA remain unclear. Additionally, there is a lack of knowledge regarding the cutoff values for reticulocyte counts that may predict the efficacy of TPO-RA treatment. Therefore, we analyzed data from the phase 2 dose-finding study in conjunction with the phase 2/3 study to explore factors predicting the efficacy of romiplostim in refractory AA.

## Patients and methods

### Patients

Eligible patients with AA refractory to IST who were included in the phase 2 dose-finding study (n = 35) [6] and the phase 2/3 study (n = 31) [7] were analyzed. Detailed inclusion/exclusion criteria have been previously reported [6, 7].

### Study design

This study was an integrated secondary analysis of the phase 2 dose-finding study conducted from January 2014 to March 2018 [6] and the phase 2/3 study conducted from February 2016 to July 2020 [7].

### Intervention

In the phase 2 dose-finding study, patients were randomly assigned to dose groups (1, 3, 6, or 10 μg/kg) for subcutaneous romiplostim once a week for 8 weeks based on their platelet count [6]. Doses were adjusted every 4 weeks (1–20 μg/kg per week) for up to a year (weeks 9–52) based on platelet response and safety. During the extension phase of the study, patients were administered romiplostim in increasing dosages every 4 weeks (1, 3, 6, 10, 13, 16, and 20 μg/kg once weekly), depending on their platelet response and safety, for a duration of 1 year (weeks 9–52). For patients who exhibited a positive platelet response from weeks 46–53, the dose titration process continued with single-step increments of 3, 6, 10, 13, 16, and 20 μg/kg once weekly for an additional 2 years, spanning weeks 53–156.

In the phase 2/3 study, romiplostim was injected subcutaneously at a constant dosage of 10 µg/kg per week for 4 weeks, then gradually increased to 5–20 µg/kg per week for weeks 5–52 [7]. Hematologic response was assessed after 53 weeks. Patients stopped treatment during weeks 53–56 but could resume romiplostim at the same dosage from week 56 onwards as part of the trial's extension phase. Patients were not allowed to receive other treatments for AA (e.g., immunosuppressive agents such as anti-thymocyte globulin [ATG], cyclosporine, and anabolic hormones) during the study. The dose of romiplostim was adjusted depending on platelet response and toxicity. Dose increases were conducted in single-step increments every 4 weeks until a platelet response was achieved. The dose was reduced by one step if the platelet count was > 200 × 10^9^/L. Romiplostim doses were tapered off, with the aim of discontinuation once trilineage hematopoiesis was achieved. Trilineage hematopoiesis was defined as a platelet count > 50 × 10^9^/L, hemoglobin (Hb) concentration > 10.0 g/dL, and neutrophil count > 1.0 × 10^9^/L maintained for 8 weeks with the same romiplostim dose without transfusion.

### Outcomes

Outcomes included in this analysis were as follows: patient characteristics (sex, age, disease duration, severity of AA, prior treatment history, transfusion); baseline parameters (reticulocyte count, TPO concentration, ferritin, serum iron, total iron-binding capacity, unsaturated iron-binding capacity, transferrin saturation, aspartate transaminase, alanine transaminase, platelet count, hemoglobin concentration, neutrophil count); and hematologic response (responder/ non-responder). Responders were defined as those with complete response (CR) + partial response (PR); non-responders were defined as those with no response (NR) (Table 1).

**Table 1:** Definition of hematological response

	Severity at baseline	
	Patients with SAA/VSAA	Patients with NSAA
Complete response	All of the following criteria are met: • Hb concentration ≥ 10 g/dL • Neutrophil count ≥ 1.0 × 10^9^/L • Platelet count ≥ 100 × 10^9^/L	
Partial response	Platelet and erythrocyte transfusion independence, and two or more of the following criteria are met: • Neutrophil count ≥ 0.5 × 10^9^/L • Platelet count ≥ 20 × 10^9^/L • Reticulocyte count ≥ 20 × 10^9^/L	Transfusion independence^a^ (if previously dependent) or doubling or normalization of at least one cell line or increase of baseline: • Hb > 3.0 g/dL (if initially < 6.0 g/dL) • Neutrophil count > 0.5 × 10^9^/L (if initially < 0.5 × 10^9^/L) • Platelet count > 20 × 10^9^/L (if initially < 20 × 10^9^/L)
No response	No longer meeting the above criteria	

^a^Regarding the definition of transfusion-dependent/-independent for each analysis in the phase 2 dose-finding study, the following cases were transfused

・Platelet transfusion due to platelet count < 10 × 10^9^/L (prophylactic transfusion) or bleeding (therapeutic transfusion)

・Red blood cell transfusion due to Hb concentration < 9.0 g/dL or symptomatic anemia

Definitions of transfusion-dependent and -independent in the analysis of ‘improvement of blood cell indices’ were as follows: for both platelet and red blood cell transfusions, patients were considered as transfusion-dependent if transfusion was performed within 8 weeks (56 days) prior to each time point, and transfusion-independent if transfusion was not performed for more than 8 weeks (56 days)

Definitions of transfusion-dependent and -independent in the analysis of ‘efficacy criteria’ were as follows: patients were considered as transfusion-dependent if platelet transfusion was performed within 4 weeks (28 days) prior to each time point, and considered as transfusion-independent if transfusion was not performed for more than 4 weeks (28 days); patients were considered as transfusion-dependent if red blood cell transfusion was performed within 8 weeks (56 days) prior to each time point, and considered as transfusion-independent if transfusion was not performed for more than 8 weeks (56 days)

*Hb* Hemoglobin, *NSAA* Non-severe aplastic anemia, *SAA* Severe aplastic anemia, *VSAA* Very severe aplastic anemia

### Endpoints

The primary endpoint was the identification of factors affecting the response rate at 27 and 53 weeks. CR + PR was used to define response. The definition of response in non-severe AA was the same as that described in the AA guidelines [11]. The definition of response in severe AA was based on the criteria described in the AA guidelines and modified (similar to the criteria used in the phase 2/3 study of romiplostim in IST-naive AA).

The secondary endpoint was the calculation of cutoff values for reticulocyte counts that predict response (CR + PR).

### Statistical analysis

In this study, logistic regression analysis was performed with responder/non-responder as the objective variable for baseline patient characteristics and baseline parameters. Baseline reticulocyte count as the explanatory variable was used to generate receiver operating characteristic (ROC) curves, and the Youden index was used to examine cutoff values for reticulocyte count area under the curve (AUC). The 95% confidence interval (CI) for the AUC was also calculated. Comparing the responder and non-responder groups, the chi-square and Fisher’s exact tests were used for categorical variables, and the unpaired t-test or Mann–Whitney U-test was used for continuous variables (multiplicity was not adjusted for in the present study because of the exploratory nature of the analysis). Variables were 1) patient characteristics, and 2) baseline parameters. Statistical analysis was conducted using SAS Ver.9.4 (SAS Institute Inc., Cary, NC, USA).

## Results

### Patient characteristics

The baseline characteristics of the 66 patients (35 from the phase 2 dose-finding study and 31 from the phase 2/3 study) are shown in Table 2. Overall, 60.6% of patients were female (51.4% in the phase 2 dose-finding study and 71.0% in the phase 2/3 study); median age was 47.0 (range 20–78) years. The median disease duration was 121.0 (range 1–438) months. Approximately half of the patients had non-severe AA (51.5%), followed by severe AA (39.4%), and very severe AA (9.1%). In the phase 2 dose-finding study, all patients had been previously treated with ATG + cyclosporine and 80% had received other drugs. Patients in the phase 2/3 study had previously received ATG + cyclosporine most frequently (71.0%), followed by anabolic steroids (67.7%), others (35.5%), and cyclosporine (25.8%). At baseline, 80.3% of patients were transfusion-dependent.

**Table 2:** Baseline characteristics

		Phase 2 dose-finding study (N = 35)	Phase 2/3 study (N = 31)	Total (N = 66)
Sex	Male	17 (48.6%)	9 (29.0%)	26 (39.4%)
	Female	18 (51.4%)	22 (71.0%)	40 (60.6%)
Age, years	Median (min, max)	49.0 (28, 76)	46.0 (20, 78)	47.0 (20, 78)
Disease duration, months	Median (min, max)	127.0 (9, 438)	104.0 (1, 408)	121.0 (1, 438)
Severity of AA	VSAA	2 (5.7%)	4 (12.9%)	6 (9.1%)
	SAA	17 (48.6%)	9 (29.0%)	26 (39.4%)
	NSAA	16 (45.7%)	18 (58.1%)	34 (51.5%)
Prior treatment history	ATG	0 (0.0%)	0 (0.0%)	0 (0.0%)
	CsA	0 (0.0%)	8 (25.8%)	8 (12.1%)
	ATG + CsA	35 (100.0%)	22 (71.0%)	57 (86.4%)
	Anabolic steroid	-	21 (67.7%)	21 (67.7%)
	Other	28 (80.0%)	11 (35.5%)	39 (59.1%)
Transfusion-dependent/-independent^a^	Dependent	30 (85.7%)	23 (74.2%)	53 (80.3%)
	Independent	5 (14.3%)	8 (25.8%)	13 (19.7%)

Data are n (%) unless stated otherwise

^a^Red blood cell and/or platelet transfusion: red blood cell transfusion-dependent: transfusion within 8 weeks (56 days) of enrollment; platelet transfusion-dependent: transfusion within 8 weeks (56 days) of enrollment

*AA* Aplastic anemia, *ATG* Anti-thymocyte globulin, *CsA* Cyclosporine, *NSAA* Non-severe aplastic anemia, *SAA* Severe aplastic anemia, *VSAA* Very severe aplastic anemia

### Study endpoints

Response rates are depicted in Table 3. At 27 weeks, response was seen in 35 (57.4%) patients, all of which were PR. At 53 weeks, response was seen in 34 (75.6%) patients, with CR in two (4.4%) and PR in 32 (71.1%). Red blood cell transfusion-independence (baseline: n = 49) was seen in 24 (49.0%) patients at 27 weeks, and in 22 (44.9%) at 53 weeks. Platelet transfusion-independence (baseline: n = 37) was seen in 21 patients (56.8%) at 27 weeks, and in 16 (43.2%) at 53 weeks. Univariate logistic regression analysis identified the duration from diagnosis (*P* = 0.040), baseline reticulocyte count (*P* < 0.001), and platelet count (*P* < 0.001) as factors predicting response to romiplostim at 27 weeks (Table 4). As a result of the univariate analysis of efficacy at Week 53, a higher percentage of very severe/severe AA (VSAA/SAA) was observed in the NR group (72.7%) compared with non-severe AA (27.3%), with a significant difference (*P* = 0.038) (Online Resource 1).

**Table 3:** Outcome measures: response rates and transfusion independency

**Response rates**		
Timepoint	Response rates	n (%)
27 weeks (n = 61)	Response rate: CR + PR	35 (57.4)
	CR	0 (0.0)
	PR	35 (57.4)
	NR	26 (42.6)
53 weeks (n = 45)	Response rate: CR + PR	34 (75.6)
	CR	2 (4.4)
	PR	32 (71.1)
	NR	11 (24.4)
**Transfusion independency**		
Transfusion at baseline	Timepoint	Transfusion-independent,n (%)
RBC (n = 49)	27 weeks	24 (49.0)
	53 weeks	22 (44.9)
Platelets (n = 37)	27 weeks	21 (56.8)
	53 weeks	16 (43.2)

*CR* Complete response, *NR* No response, *PR* Partial response, *RBC* Red blood cell

**Table 4:** Univariate analysis of baseline demographic variables for the prediction of response to romiplostim at 27 weeks

Factors	Category	Responder (CR + PR) (n = 35)	NR (n = 26)	Odds ratio	P^*^
Sex	Male	13 (37.1%)	11 (42.3%)	0.806	0.683
	Female	22 (62.9%)	15 (57.7%)		
Age, years (median: 47.0)	≥ median	15 (42.9%)	16 (61.5%)	0.469	0.152
Disease duration, months (median: 121.0)	< median	21 (60.0%)	9 (34.6%)	0.328	0.040
Severity of AA	VSAA/SAA	13 (37.1%)	16 (61.5%)	0.369	0.062
	NSAA	22 (62.9%)	10 (38.5%)		
Prior treatment history	ATG + Cyclosporine	28 (80.0%)	24 (92.3%)	0.389	0.274
	Cyclosporine	6 (17.1%)	2 (7.7%)		
Reticulocyte count	≥ median	25 (71.4%)	6 (23.1%)	8.333	< 0.001
(median: 37.77 × 10^9^/L)	< median	10 (28.6%)	20 (76.9%)		
Hb concentration^a^	≥ median	15 (42.9%)	5 (19.2%)	1.286	0.724
(median: 6.75 g/dL)	< median	14 (40.0%)	6 (23.1%)		
Neutrophil count	≥ median	21 (60.0%)	10 (38.5%)	2.400	0.099
(median: 0.84 × 10^9^/L)	< median	14 (40.0%)	16 (61.5%)		
Platelet count	≥ median	27 (77.1%)	5 (19.2%)	14.172	< 0.001
(median: 10.0 × 10^9^/L)	< median	8 (22.9%)	21 (80.8%)		

Data are n (%) unless stated otherwise

^*^ Fisher’s exact test

^a^For Hb concentration, the total number was 29 patients for CR + PR and 11 patients for NR due to some patients with missing data

*AA* Aplastic anemia, *ATG* Anti-thymocyte globulin, *CR* Complete response, *Hb* Hemoglobin, *NR* No response, *NSAA* Non-severe aplastic anemia, *PR* Partial response, *SAA* Severe aplastic anemia, *VSAA* Very severe aplastic anemia

Online Resource 2 summarizes the additional baseline parameters and logistic regression analysis per response groups using these baseline values. The TPO levels showed higher mean values in the NR group (2996.9 pg/ml) compared with the responder (CR + PR) group (2348.1 pg/ml), though the difference was not statistically significant (*P* = 0.136). Ferritin levels were higher in the NR group (mean 2115.50 ng/ml) compared with the responder group (mean 1409.37 ng/ml), but this difference was also not statistically significant (*P* = 0.220). Serum iron levels were significantly higher in the NR group (mean 260.3 µg/dL) compared with the responder group (mean 213.4 µg/dL) (*P* = 0.029). Total iron binding capacity levels were also significantly higher in the NR group (mean 385.1 µg/dL) compared with the responder group (mean 308.4 µg/dL) (*P* = 0.043). Other factors, including unsaturated iron binding capacity, transferrin saturation, aspartate aminotransferase, and alanine transaminase, did not show statistically significant differences between the two groups. Additionally, the median baseline TPO concentration was 2375.0 pg/mL for responders and 2680.0 pg/mL for non-responders, with no significant differences (Online Resource 3).

**Fig. 1:**
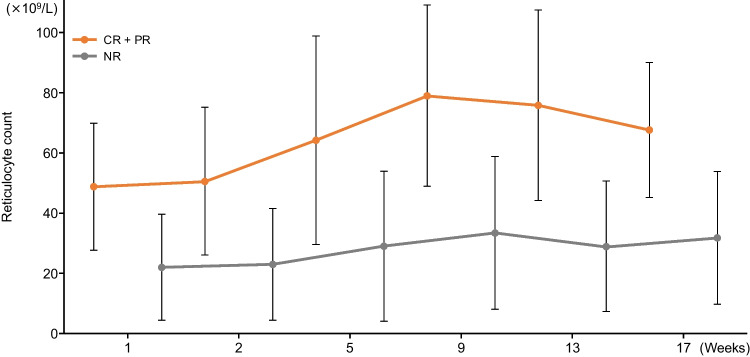
Reticulocyte counts (mean ± SD) according to responders and non-responders. *CR* Complete response, *NR* No response, *PR* Partial response, *SD* Standard deviation

**Fig. 2:**
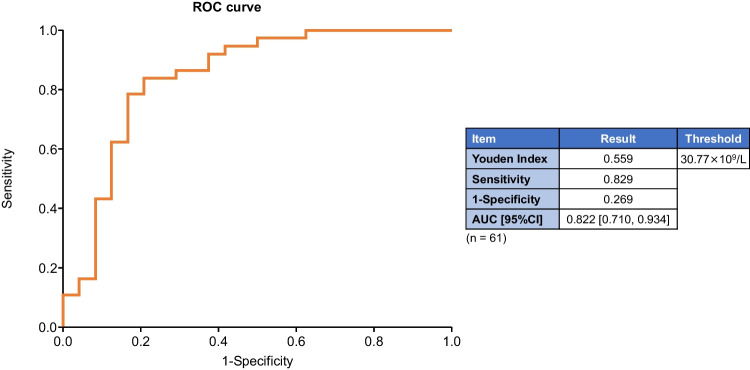
ROC analysis results: reticulocyte count thresholds. The figure shows the efficacy criteria (CR + PR, NR) and reticulocyte count thresholds at 27 weeks. *AUC* Area under the curve, *CI* Confidence interval, *CR* Complete response, *NR* No response, *PR* Partial response, *ROC* Receiver operating characteristic

The time course of reticulocyte counts is shown in Fig. 1. Counts were significantly higher for responders (CR + PR) compared those with NR throughout the period (logistic regression analysis with CR + PR and NR as response variables). In addition, ROC curve analysis calculated AUC and cutoff values for reticulocyte count relative to response rate (CR + PR, NR) at 27 weeks as follows: AUC 0.822 [95% CI 0.710, 0.934], threshold 30.77 × 10^9^/L (sensitivity: 82.9%, specificity: 73.1%) (Fig. 2). This cutoff value was predictive of response to romiplostim.

**Fig. 3:**
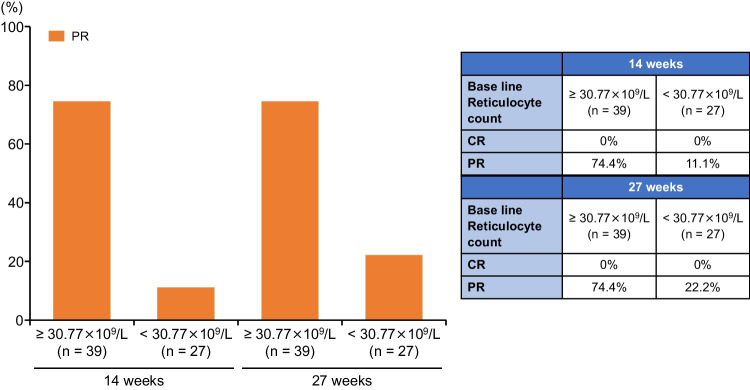
Response rates by baseline reticulocyte count threshold. *CR* Complete response, *PR* Partial response

Response rates by baseline reticulocyte count threshold (≥ 30.77 × 10^9^/L, < 30.77 × 10^9^/L) were 74.4% and 11.1%, respectively, for PR at 14 weeks, and 74.4% and 22.2%, respectively, for PR at 27 weeks (Fig. 3).

## Discussion

The present study aimed to identify factors that predict hematologic response to romiplostim in AA patients who do not respond to IST. It also explored reticulocyte count as an efficacy indicator for romiplostim using data from the phase 2 and phase 2/3 studies of romiplostim in patients with refractory AA. Although the phase 2 and phase 2/3 studies in refractory AA evaluated response by each cell lineage (erythrocyte, platelet, and neutrophil response), the present study evaluated response according to the CR/PR/NR definitions, which are described in guidelines [11] and commonly used in general practice. In the present study, no patients achieved CR at 27 weeks. This is because in the phase 2 and phase 2/3 studies, the doses of romiplostim were increased or decreased based on platelet counts, and the dosing approach was not targeted at achieving CR.

The main finding of this study was the identification of factors that predict response to romiplostim treatment for patients with AA. Univariate logistic regression analysis revealed that disease duration (*P* = 0.040), reticulocyte count (*P* < 0.001), and platelet count (*P* < 0.001) were potentially predictive of response. In addition, the reticulocyte count was significantly higher in responders (CR + PR) compared with that in non-responders (NR) throughout the period by logistic regression analysis with CR + PR and NR as response variables. The baseline reticulocyte count of 30.77 × 10^9^/L was used as the cutoff value to predict the response rate. However, multivariate analysis was not performed in the present study. The first reason for this is that the results of univariate analysis include factors (platelet count and neutrophil count) that have a strong interaction with response (CR + PR), which may lead to misleading results in multivariate analysis. The second reason is that the number of patients in this study (CR + PR: 35 patients, NR: 26 patients) was too small to perform multivariate logistic regression analysis, making it difficult to set explanatory variables. Because of the small number of patients in this study, forcing the addition of multiple factors into multivariate analysis is likely to result in either incorrect estimates or results with poor reproducibility whose estimates apply only to the data used in the present analysis (i.e., overfitting).

The importance of reticulocyte count as a predictor of TPO-RA response in AA has been suggested. In the present study using ROC curves, a cutoff value of 30.77 × 10^9^/L for the reticulocyte count was predictive of response rate, whereas in a previous study, predictive factors for response to eltrombopag for refractory AA were absolute reticulocyte count and duration from the first IST [8]. In a study of romiplostim in patients with refractory AA with inadequate response to eltrombopag, predictive factors for response were absolute reticulocyte count, neutrophil count, and hemoglobin level at baseline [12]. In the present study, the lack of a significant difference in median baseline TPO concentration between responders and non-responders (2375.0 pg/mL and 2680.0 pg/mL, respectively) suggests that baseline TPO levels may not predict treatment response, whereas a previous study of IST + eltrombopag in untreated patients showed that TPO concentrations were significantly lower in responders. [10] This lack of similarity may be because data on TPO concentrations were available for only 32 cases with response rate, and because of the different treatment settings between studies (frontline vs salvage treatment).

The present study has several limitations. First, the results of the two trials were combined and analyzed, and because the phase 2 dose-finding study had a dose-setting phase, it included fewer patients who used the starting dose in actual clinical practice. Second, factors that may predict response were not examined in the multivariate analysis, and the effects between variables may not have been properly adjusted. Thus, the results should be interpreted with caution. Third, this study was based on studies that included Asian populations; therefore, generalizability to non-Asian populations is limited.

In conclusion, logistic regression analysis showed that, among baseline demographic variables, disease duration, reticulocyte count, and platelet count were possible predictors of response to romiplostim. A cutoff value of 30.77 × 10^9^/L for the reticulocyte count was predictive of response to romiplostim treatment in patients with refractory AA. In addition, the present analysis includes fewer cases treated with the starting dose used in actual clinical practice. Therefore, even in patients with a baseline reticulocyte count < 30.77 × 10^9^/L, it is possible that response could be achieved by increasing the dose of romiplostim early to the maximum dose of 20 µg/kg in patients with refractory AA, who otherwise have limited therapeutic options.

## Supplementary Information

Below is the link to the electronic supplementary material.

Supplementary file1 (DOCX 51.4 KB)

## Data Availability

The datasets used in the current analysis are available from the corresponding author upon reasonable request.

## Declarations

In the phase 2 dose-finding study, the ethical considerations were as follows: approval by the Ethics Review Board, adherence to the Declaration of Helsinki, provision of informed consent by all patients at enrollment, and clinical trial registration at ClinicalTrials.gov (NCT02094417). In the phase 2/3 study, the ethical considerations were as follows: approval by the Institutional Review Board at each center, adherence to the Declaration of Helsinki and International Conference on Harmonisation of Good Clinical Practice guidelines, provision of informed consent by all participants, and clinical trial registration at ClinicalTrials.gov (NCT02773290). Consent for secondary use of the data in this study was also obtained.
