# Supplementary material for: Predictive factors of romiplostim response in patients with refractory aplastic anemia: data from two clinical trials
Source: Ann Hematol. 2025 Jul 1;104(8):4003–11. doi: 10.1007/s00277-025-06337-7 (PMC12431918; doi:10.1007/s00277-025-06337-7)
Supplement: Supplementary file 1 — Supplementary file1 (DOCX 51.4 KB) [file 277_2025_6337_MOESM1_ESM.docx]

*Annals of Hematology*

# **Predictive factors of romiplostim response in patients with refractory aplastic anemia: data from two clinical trials**

Jun Ho Jang, Kinuko Mitani, Yoshiaki Tomiyama, Koji Miyazaki, Koji Nagafuji, Kensuke Usuki, Nobuhiko Uoshima, Tomoaki Fujisaki, Hiroshi Kosugi, Itaru Matsumura, Ko Sasaki, Masahiro Kizaki, Masashi Sawa, Michihiro Hidaka, Naoki Kobayashi, Satoshi Ichikawa, Yuji Yonemura, Kenta Murotani, Mami Shimizu, Akira Matsuda, Keiya Ozawa, Shinji Nakao, and Jong Wook Lee

**Online Resource 1.** Comparison of demographic and clinical characteristics between responder and NR groups at 53 weeks

| Factors | Category | Responder (n = 34) | NR (n = 11) | Odds ratio | *P*^*^ |
| --- | --- | --- | --- | --- | --- |
| Sex | Male | 13 (38.2%) | 3 (27.3%) | 1.650 | 0.512 |
|  | Female | 21 (61.8%) | 8 (72.7%) |  |  |
| Age, years  (median: 47.0) | ≥ median | 17 (50.0%) | 7 (63.6%) | 0.572 | 0.434 |
| Disease duration, months  (median: 121.0) | < median | 19 (55.9%) | 3 (27.3%) | 0.296 | 0.109 |
| Severity of AA | VSAA/SAA | 12 (35.3%) | 8 (72.7%) | 0.205 | 0.038 |
|  | NSAA | 22 (64.7%) | 3 (27.3%) |  |  |
| Prior treatment history | ATG + CsA | 26 (76.5%) | 10 (90.9%) | 0.325 | 0.317 |
|  | CsA | 8 (23.5%) | 1 (9.1%) |  |  |
| Reticulocyte count (median: 40.48 × 10^9^/L) | ≥ median | 22 (64.7%) | 1 (9.1%) | 18.327 | 0.0087 |
|  | < median | 12 (35.3%) | 10 (90.9%) |  |  |
| Hb concentration^a^ (median: 6.80 g/dL) | ≥ median | 15 (44.1%) | 1 (9.1%) | 2.308 | 0.514 |
|  | < median | 13 (38.2%) | 2 (18.2%) |  |  |
| Neutrophil count (median: 0.84 × 10^9^/L) | ≥ median | 19 (55.9%) | 4 (36.4%) | 2.217 | 0.266 |
|  | < median | 15 (44.1%) | 7 (63.6%) |  |  |
| Platelet count (median: 12.0 × 10^9^/L) | ≥ median | 20 (58.8%) | 3 (27.3%) | 3.809 | 0.079 |
|  | < median | 14 (41.2%) | 8 (72.7%) |  |  |

Data are n (%) unless stated otherwise.

^*^ Fisher’s exact test

^a^ For Hb concentration, the total number was 29 patients for responder (CR + PR) and 11 patients for NR due to some patients with missing data.

*AA* Aplastic anemia, *ATG* Anti-thymocyte globulin, *CR* Complete response, *CsA* Cyclosporin A, *Hb* Hemoglobin, *NR* No response, *NSAA* Non-severe aplastic anemia, *PR* Partial response, *SAA* Severe aplastic anemia, *VSAA* Very severe aplastic anemia

**Online Resource 2.** Comparison of hematologic parameters between responder (CR + PR) and NR Groups

| Factors | Category | Responder | NR | *P** |
| --- | --- | --- | --- | --- |
| TPO (pg/ml) | No. of patients | 16 | 16 |  |
|  | Mean (SD) | 2348.1 (653.1) | 2996.9 (1358.3) | 0.136 |
|  | Median (min, max) | 2375.0 (1000, 3380) | 2680.0 (1860, 7100) |  |
| Ferritin (ng/ml) | No. of patients | 37 | 24 |  |
|  | Mean (SD) | 1409.37 (1884.48) | 2115.50 (2367.27) | 0.220 |
|  | Median (min, max) | 880.10 (16.8, 10724.2) | 1624.65 (330.5, 10227.5) |  |
| Serum iron  (㎍/dL) | No. of patients | 37 | 24 |  |
|  | Mean (SD) | 213.4 (88.0) | 260.3 (47.5) | 0.029 |
|  | Median (min, max) | 223.0 (16, 491) | 259.0 (189, 355) |  |
| TIBC (㎍/dL) | No. of patients | 36 | 24 |  |
|  | Mean (SD) | 308.4 (88.6) | 385.1 (175.9) | 0.043 |
|  | Median (min, max) | 287.0 (176, 558) | 308.5 (189, 868) |  |
| UIBC (㎍/dL) | No. of patients | 37 | 24 |  |
|  | Mean (SD) | 93.5 (93.9) | 125.4 (146.7) | 0.303 |
|  | Median (min, max) | 45.0 (1, 333) | 55.5 (0, 513) |  |
| TSAT (%) | No. of patients | 36 | 24 |  |
|  | Mean (SD) | 70.999 (25.716) | 75.687 (20.836) | 0.453 |
|  | Median (min, max) | 80.664 (4.58, 99.55) | 81.125 (37.92, 100.00) |  |
| AST (U/L) | No. of patients | 37 | 24 |  |
|  | Mean (SD) | 24.6 (12.6) | 22.1 (15.8) | 0.497 |
|  | Median (min, max) | 22.0 (12, 66) | 18.5 (10, 92) |  |
| ALT (U/L) | No. of patients | 37 | 24 |  |
|  | Mean (SD) | 28.7 (24.5) | 19.3 (9.5) | 0.101 |
|  | Median (min, max) | 18.0 (7, 101) | 17.5 (7, 49) |  |

* *P* value for logistic regression analysis with responder (CR+PR) and NR as response variables. *ALT* Alanine transaminase, *AST* Aspartate aminotransferase, *CR* Complete response, *NR* No response, *PR* Partial response, *SD* Standard deviation, *TIBC* Total iron binding capacity, *TPO* Thrombopoietin, *TSAT* Transferrin saturation, *UIBC* Unsaturated iron binding capacity

**Online Resource 3.** TPO concentration by responses (CR+PR vs NR)


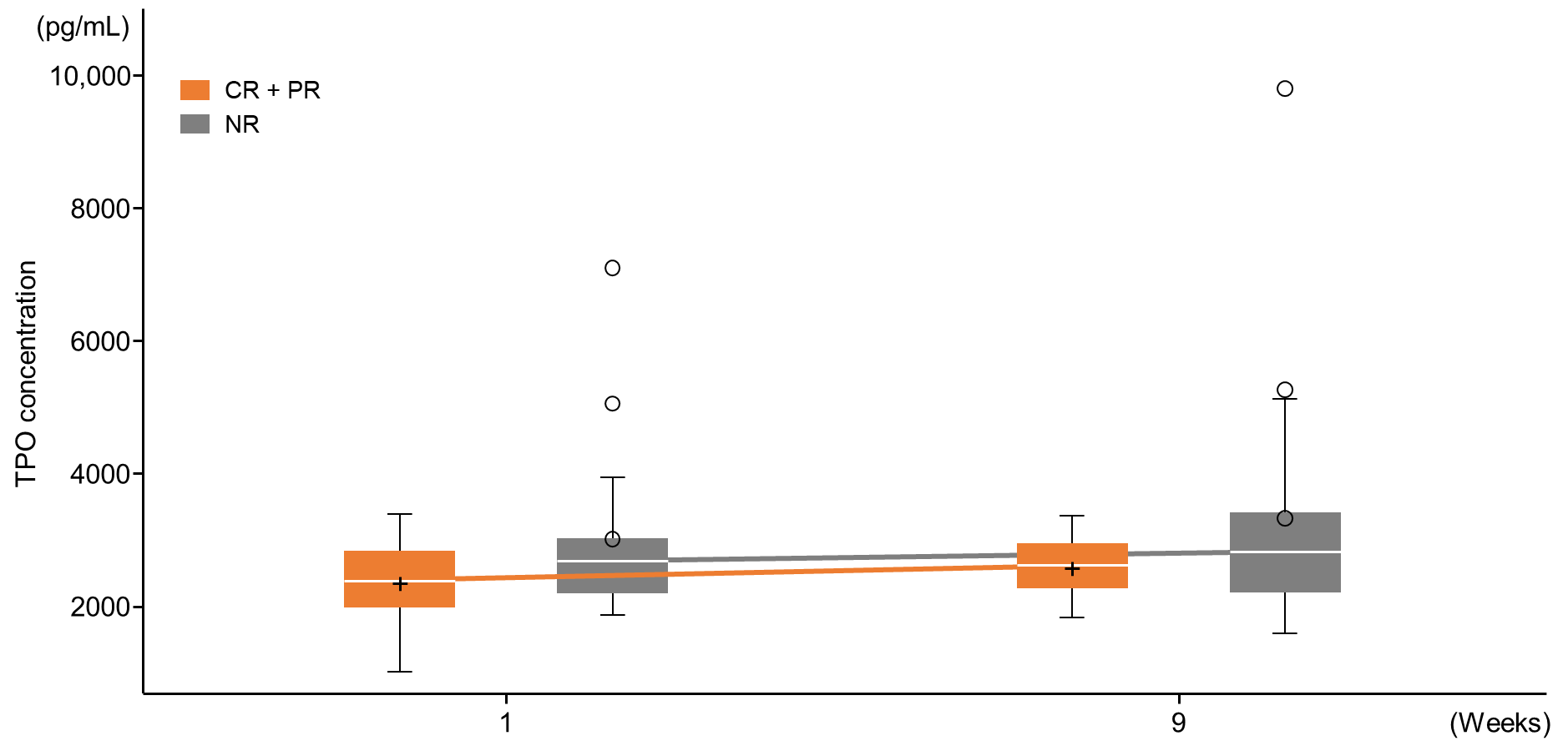


*CR* Complete response, *NR* No response, *PR* Partial response, *TPO* Thrombopoietin
